# A process for creating data report-back tools to improve equity in environmental health

**DOI:** 10.1186/s12940-022-00880-w

**Published:** 2022-07-12

**Authors:** Kathryn S. Tomsho, Erin Polka, Stacey Chacker, David Queeley, Marty Alvarez, Madeleine K. Scammell, Karen M. Emmons, Rima E. Rudd, Gary Adamkiewicz

**Affiliations:** 1 Department of Environmental Health, Harvard T.H. Chan School of Public Health, 02215 Boston, MA USA; 2 Department of Environmental Health, Boston University School of Public Health, 02118 Boston, MA USA; 3 Health Resources in Action, 02116 Boston, MA USA; 4 Mystic River Watershed Association, 02476 Arlington, MA USA; 5 Department of Social and Behavioral Sciences, Harvard T.H. Chan School of Public Health, 02115 Boston, MA USA

**Keywords:** Data report-back, Environmental health literacy, Health literacy, Materials assessment, Formative research

## Abstract

**Background:**

Although there is increasing interest in reporting results of environmental research efforts back to participants, evidence-based tools have not yet been applied to developed materials to ensure their accessibility in terms of literacy, numeracy, and data visualization demand. Additionally, there is not yet guidance as to how to formally assess the created materials to assure a match with the intended audience.

**Methods:**

Relying on formative qualitative research with participants of an indoor air quality study in Dorchester, Massachusetts, we identified means of enhancing accessibility of indoor air quality data report-back materials for participants. Participants (*n* = 20) engaged in semi-structured interviews in which they described challenges they encountered with scientific and medical materials and outlined written and verbal communication techniques that would help facilitate engagement with and accessibility of environmental health report-back materials. We coupled these insights from participants with best practice guidelines for written materials by operationalizing health literacy tools to produce accessible audience-informed data report-back materials.

**Results:**

The resulting data report-back materials had a 7th -grade reading level, and between a 4th -8th grade level of overall document complexity. The numeracy skills required to engage with the material were of the lowest demand, and we incorporated best practices for risk communication and facilitating understanding and actionability of the materials. Use of a rigorous assessment tool provides evidence of accessibility and appropriateness of the material for the audience.

**Conclusions:**

We outline a process for developing and evaluating environmental health data reports that are tailored to inspire risk-reduction actions, and are demonstrably accessible in terms of their literacy, numeracy, and data visualization demand. Adapting health literacy tools to create and evaluate environmental data report-back materials is a novel and evidence-based means of ensuring their accessibility.

**Supplementary Information:**

The online version contains supplementary material available at 10.1186/s12940-022-00880-w.

## Introduction

Participants of research studies contribute their time, environmental and/or biological samples voluntarily while accepting potential risks to their own wellbeing [1]. The information gained from participants is critical for improving understanding of causes of adverse health outcomes and for identifying public health interventions [1]. Within the last decade, interest in returning back results to participants in research studies has dramatically increased [1]. Despite early concerns of causing distress or anxiety for participants, ethical motivations for returning research results are compelling: democratizing knowledge, motivating action to improve health outcomes, and informing decision-making [2–5].

Data democratization, or the act of making information traditionally available to select groups accessible to many, provides a meaningful opportunity for information and power sharing through co-created knowledge [6]. Environmental exposure assessment data can provide meaningful insights into individuals’ exposures in their personal lives that they would not otherwise be able to characterize due to the need for technical expertise and specialized equipment. The information can help participants make very practical and applied choices about how they live their lives, such as avoiding certain products or changing certain behaviors [5, 7–11]. However, the utility of the data report-back only exists when data are presented in an accessible and appropriate manner. Providing data back in a way that is jargon-laden, or incomprehensible to some participants is an incomplete sharing of the data and power of information. Environmental data reports that demand high literacy or numeracy skills risk perpetuating data inequities and health disparities by only facilitating data understanding and motivating action in those with more advanced literacy and numeracy skills [7, 11, 12]. Several broad-scale surveys indicate that literacy and numeracy skills are limited among adults in the United States [13–16]. Ensuring broad accessibility of environmental data reports is paramount to ensuring equitable utility for all participants, regardless of educational background, literacy/numeracy skills, or formal training.

Increased interest in the data report-back in exposure assessment studies, combined with growing emphasis on community engagement and participatory research, have prompted efforts to better define best practices for environmental data report-back [2, 3, 8, 17–23]. One such effort included a workshop at the 2018 Partnerships in Environment and Public Health (a National Institute of Environmental Health Sciences program comprised of scientists, educators, health care providers, community-members, and policy makers) conference, in which participants were asked to identify issues and priorities among community-engaged researchers to build the report-back foundations and improve the process for material recipients [24, 25]. Cluster mapping of 35 attendees’ responses identified five domains of areas for improvement for environmental data report-back [24].

These five domains provide a roadmap for environmental health communicators to address knowledge gaps in the process of providing data and materials back to research participants. In particular, there was consensus that a primary priority should be determining ways of “communicating information to study participants to ensure they understand what it means” [24]. To meet this goal, communication developers must create materials that are accessible and appropriate for the intended audience. This necessitates an understanding of the audience, their literacy and numeracy skills, and creating materials to suit their existing skill sets. We describe here a research-driven approach that combines formative research with health literacy tools to tailor materials to communicate indoor air quality data back to participants in way that was sensitive to their experience with indoor air quality.

One of the goals in sharing data with study participants is to provide information that is useful for making decisions about their own environment [22]. This involves a stimulus (the data report), processing and meaning made by the reader, memory of the materials, and the outcome of action or inaction. Fuzzy-trace theory describes the process of judgement (processing and meaning) and decision making (outcome) based on memory and reasoning from a stimulus [26]. The theory distinguishes between two types of memory processing practices, gist and verbatim, to categorize the ways in which people, “encode, store, retrieve, and forget” memory information [26].

Gist-level refers to the essential information, or “bottom-line meaning” of the material [27]. Verbatim memory refers to the precise quantitative aspects of information, and encapsulates the exact form in which the information was presented [27]. When making decisions, particularly related to risk, people tend to rely on their gist memory (rather than verbatim) to act [27]. Thus, gist representations of the data facilitate better understanding of risk and decision-making [28]. Gist-level representations, particularly visual demonstrations, are associated with better long-term decision making specifically in the context of environmental health risks [29].

Although gist-level information can facilitate decision-making, gist meaning is intuitive and requires background knowledge or prior experience with the topic of interest [30, 31]. This background information impacts the encoding of the new information as extraction of the bottom-line meaning [30, 31]. Thus, people who have more training or experience with a given topic will more accurately apply gist-level information to assess risk [30, 31]. An investigation on how to build gist-level understanding looked at how experts vs. laypersons interpret quantitative risk information and found that, “numbers should be presented so that people can extract their own gist, but that they should be ordered and organized to facilitate recognition of patterns and basic meaning, with explicit labels summarizing the bottom line as judged by experienced patients and providers” [30]. Thus, providing verbatim-level representations of the quantitative information or data coupled with facilitators for interpretation can assist with building intuition for contextualizing the data and associated risk necessary to build gist-level understanding.

This effort provides guidance for environmental health communicators as they create accessible report-back materials, and methods to address many of the themes identified as priority areas of improvement for environmental health report-back.

## Methods

### Formative research

 Formative research provides critical insight as to how to best tailor communications for a specific audience [23, 24]. Formative research typically occurs early in the communication development process so that information can be collected on audience characteristics and understanding, as well as contextual factors that are pertinent to the message [32]. This information is collected early in the communication creation process to inform the development and tailoring of the message to be appropriate and accessible for the intended audience [33]. Formative research has been a critical tool for development of appropriate health-related communications and tailoring them for recipients with varied health literacy skills [12, 32, 34, 35].

#### Study population

 This report-back effort reflects the joint work of the Home-based Observation and Monitoring Exposure (HOME) Study and Community Engagement Core members within the Center for Research on Environmental and Social Stressors in Housing across the Life Course (CRESSH) [36]. The HOME Study collected indoor air quality data in the homes of 78 participants living in Dorchester, Massachusetts. Data collection began in the summer of 2017 and was completed in summer of 2019. Nitrogen dioxide (NO_2_) and particulate matter particles smaller than or at 2.5 micrometers (PM_2.5_) data were reported back to participants via printed materials in winter of 2020. At that time, participants were invited to attend meetings with members of the research team to discuss their household data reports. Due to the onset of the COVID-19 pandemic, all participant meetings were completed virtually.

#### Interviews

 Semi-structured interviews were conducted with 20 of the HOME Study participants before creation of the report-back materials. Prior to the interviews, participants had completed both a baseline survey questionnaire about their home characteristics, demographics, and health behaviors, as well as two seasons of indoor air quality monitoring – one week each in the summer and winter seasons [37]. All participants who completed both seasons of sampling were categorized based on their responses to a question regarding their perception of Dorchester’s air quality (6 response options, ranging from ‘Very bad’ to ‘Very good’, ‘Uncertain’, or ‘I have never thought about it’). For the interviews, participants were randomly sampled from each of the categorical response bins related to air quality perception.

The first ten interviews were performed within the participants’ homes, and the final ten were performed over the phone due to the onset of the COVID-19 pandemic [38]. Each interview was approximately an hour, and covered topics included typical in-home behaviors and products, perceptions of indoor air quality in the home, prior experience with scientific and medical materials, and input specifically regarding the creation of the report-back materials for this study.

#### Qualitative data analysis

All interviews were audio-recorded at the consent of the participant. Interviews were transcribed by the first author and then entered into NVivo 12 Pro. Our full qualitative analysis of the semi-structured interviews is described elsewhere [38]. Briefly, this analysis implemented the three step approach of grounded theory: open, axial, and selective thematic coding [38]. We examined the confirmability, transferability, and credibility of the data as means of establishing rigor and trustworthiness in our qualitative analysis [38, 39]. Detailed memos were kept from each interview, and throughout the qualitative analysis process. All qualitative analysis was performed within NVivo 12 Pro.

These interviews were instrumental in characterizing participants’ current understanding of indoor air quality, prior experience with the topic, and providing insight as to what they expected from the report-back materials.

### Application of health literacy tools:

#### Step 1: Overall structure – Fuzzy Trace Theory

We developed the organization of our report-back material to facilitate both styles of meaning making for our audience. This was based on the understanding that some participants had prior experiences that led them to have background knowledge of air quality, whereas for some this topic was novel. Guidance for material development from Fuzzy Trace Theory was as follows [28–31, 40, 41]:

1. Provide bottom-line messages regarding data in consolidated form for gist-level meaning.
  - Include critical information within the gist information (i.e. risk, call-to-action, etc.).
  - Include visual summaries, such as graphics, to facilitate gist-level recall.
  - Provide brief interpretation of data meaning for participants.
2. Include verbatim information as a means of building intuition for gist-level meaning.
  - Include expert interpretations of data patterns or trends identified in the data.

#### Step 2: Operationalizing health literacy tools

We applied four evidence-driven health literacy tools to tailor our materials to be accessible for our participants in terms of the literacy, graphicacy, and numeracy demands [28, 42–45]. These tools were selected to address components of the report in a complementary manner. The Simple Measure of Gobbledygook (SMOG) was selected to aid in addressing the literacy demand, the PMOSE/IKIRSCH provided guidance to limit the complexity and density of the materials, Apter’s hierarchy provided guidance on how to limit the numeracy demand, and Visualizing Health suggested how to best communicate risk visually [46–49]. An overview of tools used, and the best practice guidance provided from each is provided below. A summary of each tool, its focus, and extracted principles is provided in Table 1.

**Table 1:** Summary of applied tools’ focuses and extracted principles

Tool	Focus	Principles Extracted
Simple Measure of Gobbledygook (SMOG) [46]	Literacy demand	1. Use short sentences. 2. Use words with fewer than three syllables where possible. 3. Use audience-friendly language (rather than jargon).
PMOSE/IKIRSCH [47]	Document complexity	1. Use few labels and items per graphic. 2. Organize labels and items simply. 3. Do not refer to information outside of the page (each page has all information needed).
Apter’s Hierarchy [48]	Numeracy demand	1. Use few mathematical constructs within the material. 2. If mathematical constructs are necessary, reduce the level of numerical mastery (decision making, interpretation, or description) required of the material user. 3. Include only numeracy elements that are critical to the communication goals of the material. 4. Include multiple interpretations (qualitative/verbal, quantitative, and visual) of the numeracy components.
Visualizing Health [49]	Visual risk communication	1. Identify the communication goal of each data visualization based on: a. The amount of detail to be conveyed (i.e., gist vs. verbatim), b. The risk message (i.e., risk tradeoffs, differences in likelihood, raising/lowering concerns, classifying risks, or awareness of risk), c. The data to be communicated (such as risk estimate or test result). 2. Employ the graphical best practices identified by Visualizing Health to tailor data visualizations to meet the outlined goals from above.

##### SMOG

The Simple Measure of Gobbledygook (SMOG) is a readability assessment tool [46]. The SMOG provides a mathematical equation to assess the reading difficulty of a passage or material based on two parameters: the length of individual words and the length of sentences [46]. The output from the SMOG is the reading grade level of the text [46]. Longer words and longer sentences present more difficulty for the reader [46]. Accordingly, to reduce the literacy demand of the text, the goal of the material creator is to reduce the length of both individual words and sentences.

To operationalize the SMOG when writing our report back materials, we implemented these three guiding principles:

1. Use short sentences.
2. Use words with fewer than three syllables where possible.
3. Use audience-friendly language (rather than jargon).

Several polysyllabic words (i.e. [air] quality, particulate matter, nitrogen dioxide, Dorchester) were critical to our material’s content. To account for these words, which appeared with frequency in our materials, we applied the SMOG to our materials in two manners: (1) counting every instance of every polysyllabic word; and (2) counting those four critical polysyllabic words listed above only the first time that they occurred in the material.

##### PMOSE/IKIRSCH

 The PMOSE/IKIRSCH was introduced in 1998 to address the lack of tools to assess comprehensive document complexity [47]. The PMOSE/IKIRSCH assesses the document structure, density, and dependency [47]. Documents’ structure are assessed based on the visual organization of information, increasing in visual difficulty from simple lists, combined lists (including pie charts and timelines), intersected lists (including bar charts, line graphs and maps), and nested lists (including bar charts and graphs with nested labels) [47]. Density of the document is scored based on the number of labels and items presented within the structure [47]. Finally, dependency is considered present within a document if there is a reference to pertinent information that the reader must search for outside of the document [47]. The PMOSE/IKIRSCH assigns a numerical score based on the combination of the document’s structure, density, and dependency, and provides a grade level range of demand on the reader to contextualize the score.

To operationalize the PMOSE/IKIRSCH when creating our materials, we implemented the following principles:

1. Use few labels and items per graphic.
2. Organize labels and items simply.
3. Do not refer to information outside of the page (each page has all information needed).

##### Apter’s Hierarchy

Our data report involved substantial numeracy components, critical to communicating household air pollutant concentrations. Numeracy elements are often key elements of environmental health data reports that provide data as a means of facilitating data ownership [8].

Although there does not yet exist a tool to assess numeracy demand, Apter et al. developed a conceptual model mapping the numeracy skills necessary for health communication [48]. Numeracy refers to a range of math skills required to engage with and act upon numerical information, including comparison of values, interpretation of trends, contextualizing probabilities, estimating risk, and the array of arithmetic operations [48]. Repeated surveys have indicated that adults in the United States have lower numeracy levels than adults in other countries, which limits their ability to engage with health-relevant information [13, 42, 48, 50, 51].

Apter’s model relies on Golbeck’s four categories of numerical information, “basic (e.g., ability to identify and read numbers), computational (e.g., counting and arithmetic), analytical (e.g., inference, estimation, proportion, percentage, frequencies, basic graphs), and statistical (e.g., basic probability, statistics, and risk assessment)” [48]. Numeracy elements are organized on the left in order of difficulty, increasing as you move towards the bottom of the model [48]. The three levels of mastery required for each numeracy element increases from left (describe) to right (decision-making) [48]. Moving a required skill from one cell in the model upwards and to the left reduces the numeracy burden on the reader [48].

While Apter’s hierarchy can be used to evaluate existing numeracy materials, it may also be operationalized as a guide to decrease numeracy demands of written materials for the user [48]. Below are the steps we implemented when developing our data reports to increase accessibility of the numerical components and reduce the numeracy demand on the reader:

1. Use few mathematical constructs within the material.
2. If mathematical constructs are necessary, reduce the level of numerical mastery (decision making, interpretation, or description) required of the material user.
3. Include only numeracy elements that are critical to the communication goals of the material.
4. Include multiple interpretations (qualitative/verbal, quantitative, and visual) of the numeracy components.

##### Visualizing health

 The Visualizing Health project tested multiple iterations of risk-based health visualizations with the general public to find the most efficacious risk communication visuals [49]. This effort identified a range of risk communication scenarios, and then identified the goals of each [49]. They evaluated a series of data designs for each scenario and evaluated which made sense to the general public via survey testing [49]. From Visualizing Health, we employed the following steps to create our data visuals.

1. Identify the communication goal of each data visualization based on:
  - The amount of detail to be conveyed (i.e., gist vs. verbatim),
  - The risk message (i.e., risk tradeoffs, differences in likelihood, raising/lowering concerns, classifying risks, or awareness of risk),
  - The data to be communicated (such as risk estimate or test result).
2. Employ the graphical best practices identified by Visualizing Health to tailor data visualizations to meet the outlined goals from above.

#### Step 3: Rigorous assessment of report-back materials

In addition to using guiding principles from health literacy tools in the material creation process, we also applied the CDC Clear Communication Index to the penultimate version of our report to determine whether it was suitable and accessible for our audience [52].

##### CDC Clear Communication Index

The CDC Clear Communication Index (The Index) is a research-based tool created for development and assessment of public communications in accordance with the Plain Writing Act of 2010 [52]. The Index is comprised of 20 items that help the message creator identify the primary communication goals and enhance the clarity of the message to facilitate understanding of the material. It may be used both as guidance for best practices in message creation as well as message evaluation. Each of the 20 items that pertain to the message evaluation receive a score of zero or one. The 20 item scores are converted into a composite score (out of a possible 100) [52]. A score of 90 or above is considered ‘passing’, with a score of 100 being ideal [52]. The Index assesses materials across seven categories: (1) main message and call to action, (2) language, (3) information design, (4) state of the science, (5) behavioral recommendations, (6) numbers, and (7) risk [52]. Three members of the research team, including a member of a community-based organization, rated the report according to The Index to evaluate its appropriateness for the audience before distribution.

## Results

A template version of the reports provided back to participants is included in the supplementary materials (Figure S1). Table 2 displays the demographics for all participants of the CRESSH HOME Study, and for those who participated in the semi-structured interviews. Those who participated in the interviews were not significantly different from those who did in terms of their race/ethnicity, educational attainment, or household income.

**Table 2:** Demographics of interviewed participants and Dorchester HOME study participants

	Total	No Interview	Interview	*p*-Value
	**(*N*** **= 78)**	**(*N*** **= 58)**	**(*N*** **= 20)**	
**Race**				0.36
White	28 (35.9%)	20 (34.5%)	8 (40.0%)	
Asian	9 (11.5%)	8 (13.8%)	1 (5.0%)	
Black or African American	27 (34.6%)	18 (31.0%)	9 (45.0%)	
Other	10 (12.8%)	9 (15.5%)	1 (5.0%)	
Missing	4 (5.1%)	3 (5.2%)	1 (5.0%)	
**Hispanic**				0.26
No, Not Hispanic	66 (84.6%)	47 (81.0%)	19 (95.0%)	
Yes, Hispanic	12 (15.4%)	11 (19.0%)	1 (5.0%)	
**Educational Attainment**				0.7
Up to high school diploma, GED	14 (17.9%)	12 (20.7%)	2 (10.0%)	
Some college or associate degree	17 (21.8%)	13 (22.4%)	4 (20.0%)	
Bachelor’s degree	17 (21.8%)	11 (19.0%)	6 (30.0%)	
Post graduate degree	29 (37.2%)	21 (36.2%)	8 (40.0%)	
Refused to answer	1 (1.3%)	1 (1.7%)	0 (0%)	
**Household Income**				0.54
Less than $20,000	22 (28.2%)	19 (32.8%)	3 (15.0%)	
$20,000 to $50,000	14 (17.9%)	11 (19.0%)	3 (15.0%)	
$50,000 to $100,000	21 (26.9%)	13 (22.4%)	8 (40.0%)	
$100,000 or more	17 (21.8%)	12 (20.7%)	5 (25.0%)	
Don’t know	1 (1.3%)	1 (1.7%)	0 (0%)	
Refused to answer	3 (3.8%)	2 (3.4%)	1 (5.0%)	

**Fig. 1:**
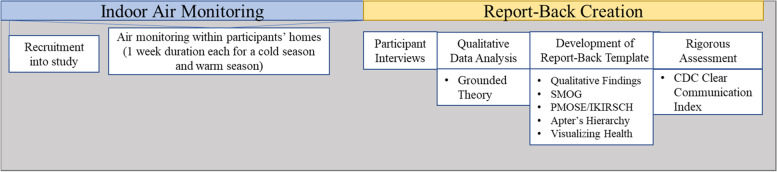
Timeline of the report-back creation process

A timeline of the overall process is displayed in Fig. 1.

We first report on the interview responses, and then how that information was used in conjunction with the health literacy tools to enhance accessibility of the materials for participants.

### Participant interview input

When asked about prior experiences with medical and/or scientific materials, participants described various barriers that they had previously encountered. The most frequently noted challenge was jargon or insufficiently explained terminology. Participants suggested that terminology which disrupted their reading flow to seek the meaning of words impacted their comprehension. In addition to jargon, ‘information overload’ and complex sentence structure were also listed as barriers.

*“…people don’t construct sentences the way they used to – that makes things plain and go the point across. Now, it seems like people just want to go on and on with a bunch of words and then at the very end you got to pick out what the important stuff really is, instead of just saying it…sometimes it’s the words that they’re using” (Participant 13)*

Participants described frustration with materials in which information was not broken down into everyday language, but instead used unfamiliar jargon. Further, they indicated that when they were unable to understand a material, they would stop engaging with it.

 Participants also identified physical and logistical components of materials that helped foster their own engagement and understanding. Providing the materials in a folder was suggested to help the recipient recognize its importance after the initial reading:

*“…sometimes you get that one piece of paper and you’re like ‘what do I do with that?’ But when they give you that nice little folder, and it says…you know a week later you read it and open it with next steps, I’m like ‘oh, I didn’t do that yet, okay let me do that now.’ So I like things that are broken down step by step. Do this, then this…when you do this, this will occur, and then the next step will be blank.” (Participant 7)*

There was also a preference to receive the materials ahead of any meeting or conversation so that participants had time to review the information and process the contents. In addition to reducing the amount of jargon and complexity of sentences, participants suggested a series of factors that would facilitate better comprehension and action based upon the report contents. First, participants asked for the materials to have an executive summary towards the front, with more detailed information behind for those who wanted to engage further. Participants suggested that breaking down actions in to small, manageable steps would make the suggestions more memorable, and more likely to be acted upon. There was also a call for these suggestions to be framed positively, rather than providing critical feedback about in-home behaviors. Finally, decision-trees were suggested as a comfortable format to help facilitate the decision-making process of what actions were appropriate for the participant to take in their home based on their personal report.

### Material layout

The final report was divided into a gist-level section in the beginning (an executive summary for each pollutant), and a verbatim-level section after. The gist-level section included the key information that participants identified as being most of interest during interviews: information about what was measured in their home, where it comes from, what the health impacts may be, whether their home’s level was of concern, and what they could do to reduce their concentrations. This information was presented numerically, graphically, and via personalized text, allowing for different learning styles to access the same information. The verbatim-level section included graphical representations of the data, charts, and personalized text and numerical components [53]. These tailored components included questions personalized to the participant’s data points and patterns. They asked participants to consider typical in-home behaviors potentially associated with the days or seasons with elevated concentrations in a workbook-style [53].

### Material complexity

Our materials included multiple data visualizations, specifically nested lists and bar charts. Each of these was scored, and all were either determined to be at Level 2 (8th grade of school equivalent), or Level 1 (4th grade of school equivalent) in terms of their complexity via the PMOSE/IKIRSCH [47]. All had few labels and items, leading them to have low material density. There was also no dependency present in any of these items. Therefore, the complexity of these items originated from the overall structure of nesting within the data tables and the inherent challenges presented by engaging with a bar chart [47].

### Literacy demand

Due to the presence of several polysyllabic words critical to our material, we determined both the overall SMOG reading level score as well as a score adjusted for the repetition of polysyllabic critical communication components. Adjusting for the repetition of these words, our final material’s literacy demand was at the 7th grade school equivalent.

### Numeracy demand

Although there is not a formal score output from Apter’s Hierarchy, we were able to decrease the numeracy demand from an early version of the report by reducing both the level of participant numeracy mastery required to engage with numerical components and using numeracy elements that were lower-demand on the reader [48]. Specifically, numerical elements included ‘reading numbers’, ‘reading tables’, and ‘reading graphs’ [48]. We further reduced the level of mastery required by providing personalized textual interpretation of the graphical and numerical components to assist with numerical interpretation [53].

We tailored our gist-level graphs (shown in Supplemental Figure S1, pages 2 & 3) in accordance with Visualizing Health’s best practices. Specifically, we aimed to classify the health risks associated with average seasonal PM_2.5_ and NO_2_ concentrations by comparing household results to the World Health Organization (WHO) indoor air guidelines [54]. To do so, we used a color-coded scheme to indicate health risk zones associated with elevated pollutant concentrations. This was also paired with personalized suggestions to refer to the specific flow-chart to help facilitate decision-making as to what actions could be taken to reduce indoor air concentrations and associated risk. Additionally, we provided interpretation of the directionality of the scale (i.e., which direction is advantageous for health) to provide additional context for the pollutant concentration results.

### Rigorous assessment

All raters assessed the materials using the CDC Clear Communication Index independently, with resulting scores of 90, 95, and 95 out of a possible 100. Raters provided notes on each detailed section during scoring, and discrepancy in scoring was compared. Score discrepancy was specifically present around whether risk was addressed both numerically and visually, however raters determined both components were present in the materials, resulting in consensus. All raters gave the materials a passing score of 90 or above, providing indication that the material is appropriate for the intended audience across these four domains.

## Discussion

A series of areas for effective communication strategies in environmental health data report-back creation have been identified: ensuring understandability of the information, communicating appropriate levels of concern/risk, how to deliver the information, defining scientific terms appropriately, visual presentation of data, and addressing health and environmental health literacy [24]. Addressing each of these domains through the combination of formative research in the form of semi-structured interviews with participants and evidence-based health literacy tools, we provide guidance for improving the process of environmental health report-back.

This effort provides specific health literacy tools that offer guidance for creating accessible materials and can be applied to assess the demand of existing report-back materials. Best practices to improve accessibility of materials were identified from each tool, and impact on report-back creation was demonstrated. There was also synergy between some of the suggestions from participants and the guidance from the health literacy tools: providing materials and low reading-grade levels, reducing sentence complexity, inclusion of an executive summary, and providing small actionable steps. This work provides both a series of steps to take to create a report-back material, as well as tools to facilitate accessibility of the material in terms of literacy, numeracy, and data visualization demand. It also provides insight as to how to facilitate action based on environmental health risk communication through message tailoring. Finally, we suggest a comprehensive tool, the CDC Clear Communication Index, as a means of evaluating the appropriateness and accessibility of a material before distribution.

### Areas for future work

There are challenges inherent in reporting-back indoor air quality monitoring data, such as: the lag between data collection, analysis, and return, identifying and communicating possible sources of pollution, and, determining the most meaningful timeframe to measure air quality in order to capture activities that may drive air pollution in the home. Additional efforts should be made to determine best practices specific to indoor air quality communication. However, the health literacy tools used in the development and assessment of these materials may be applied generally to environmental data report-back materials to enhance accessibility for intended audiences.

Providing exposure assessment data back to research study participants provides an opportunity to facilitate data democratization and enhance opportunity for informed decision-making to reduce potentially harmful exposures. However, the nature of environmental data communication necessitates inclusion of numeracy elements (such as units or scales) that may not be familiar to all participants [8]. As a result, use of numbers and data displays should be guided by current research (e.g. using whole numbers, using consistent presentation of numbers, and simplifying data visualizations) [54–57]. Additionally, the language used to contextualize and describe the results and possible health risks is often technical and unfamiliar to the broader public. Further, the nature of environmental health studies limits the ability to ethically engage in certain types of study design (such as randomized control trials), limiting conclusions that can be drawn about health risk with certainty [58]. Thus, the field of environmental health data report-back has particular hurdles in communicating findings back to the general public in a way that is accessible, appropriate, and can provide clear guidance on how to contextualize and act upon it.

Despite these challenges, developing methods to ensure accessibility of the data communication materials in a demonstrable and evidence-based manner is pivotal for equity in return of results. While tools, such as the SMOG, exist to assess and guide the reduction of literacy demand of materials, the language used within environmental exposure assessments (such as particulate matter, and nitrogen dioxide), are often polysyllabic and increase the demand on the reader. Further, these tools fail to capture short words that are unfamiliar to the audience (such as dermal), that may still obfuscate the message. While we were able to reduce the literacy demand of our materials to a 7th -grade equivalent level, meeting the national average, this level may still have been inaccessible to some members of our audience, and may not have fully captured all possible jargon [59]. In this regard, pilot testing with members of the audience is critical to ensure that the language is appropriate and understandable. This can also provide an opportunity for bi-directional decision making within the context of community-engaged research. Future work should explore whether a lower bound exists for literacy demand of environmental health communications due to necessity of polysyllabic scientific terms.

Although accessibility assessment tools, such as the CDC Clear Communication Index, exist for health-based materials, additional tools should be developed specifically for the assessment of environmental data communications. These new tools may build upon the foundation that already exists within the field of health literacy, but should incorporate environmental-health-specific considerations such as: uncertainty associated with compounds for which there is not a health-based benchmark for comparison, unclear associations between exposures and health outcomes, spatial presentation of exposure data, etc. The application of the health literacy tools within this effort provide a starting point from which future efforts may build to create comprehensive assessment instruments to evaluate and demonstrate accessibility of environmental assessment data reports.

### Limitations

 Each of the tools applied to these materials comes with their independent limitations. Using a variety of health literacy tools and best practices in concert with one another helps to overcome the individual limitations of each alone.

Although the SMOG can facilitate the reduction of literacy demand based on sentence and word length, it does not address the use of short (2 or fewer syllable) words that may limit comprehension. This leaves the possibility for vocabulary to be included in the material that is unfamiliar to the audience but not identified as challenging by the tool. The PMOSE/IKIRSCH, while helpful for structure and density of a document, does not assess the overall material beyond that (in terms of font size, appropriateness of photos, or content/vocabulary).

Apter’s Hierarchy provided guidance regarding best practices to reduce the numeracy demand on the material reader. However, this tool neither provides a numerical assessment of the demand nor does it provide explicit indication of the relationship of the numeracy skills required to engage with the material. Additional assessment of the relationship of numeracy skills required to engage with a data report-back material and the impact on health outcomes and health-based actions should be explored in future work.

The Visualizing Health project is helpful to determine appropriate visual representations of risk but does not extend to other types of non-risk visuals. Though the visuals were tested broadly to represent “ordinary individuals”, the visual communication attributes outlined may not be appropriate for specific cultural groups. Pilot testing visuals with members of the intended audience can help to ensure that the visual is communicating the intended information in an appropriate way for the recipients.

Interviews were conducted in English, limiting participation of those with other primary languages. Thus, the input from this subset of participants may not be representative of the participant pool at large. The health literacy tools used for this effort are also largely tested in English-speaking populations, and additional work regarding creation and evaluation of translated environmental health materials is needed.

Finally, due to the onset of the COVID-19 pandemic, we were unable to include a planned in-person review of the materials with a subset of participants before distribution. Feedback from participants is a critical step in generation of accessible report-backs, and should be included in the development of materials to ensure suitability for the intended audience.

## Conclusions

This effort showed the utility of using both formative research and research-based health literacy tools to create environmental health data report-backs that are demonstrably accessible and tailored to the audience. Creators of environmental data reports must acknowledge that all participants receiving communications do not come into the process with equal educational training or communication styles and skills. By reducing the demand of data communication materials, accessibility of the information within those materials may be enhanced, allowing for broader participation in the knowledge sharing. Failing to ensure accessibility of materials may disenfranchise members of the audience who have lower literacy or numeracy skills, and may perpetuate health disparities by continuing to keep environmental information out of reach for those who are vulnerable [13, 14, 60–63].

## Supplementary Information

**Additional file 1.**

## Data Availability

The datasets used and/or analyzed during the current study are available from the corresponding author on reasonable request.
