# Supplementary material for: A process for creating data report-back tools to improve equity in environmental health
Source: Environ Health. 2022 Jul 12;21:67. doi: 10.1186/s12940-022-00880-w (PMC9277935; doi:10.1186/s12940-022-00880-w)
Supplement: Supplementary file 1 — Additional file 1. [file 12940_2022_880_MOESM1_ESM.docx]

**Supplementary Materials**

Figure S1: Example of CRESSH Home Study Report

All text and numerical components in red font were tailored to each participant’s data using the MCR tool^[[1]](#footnote-1)^


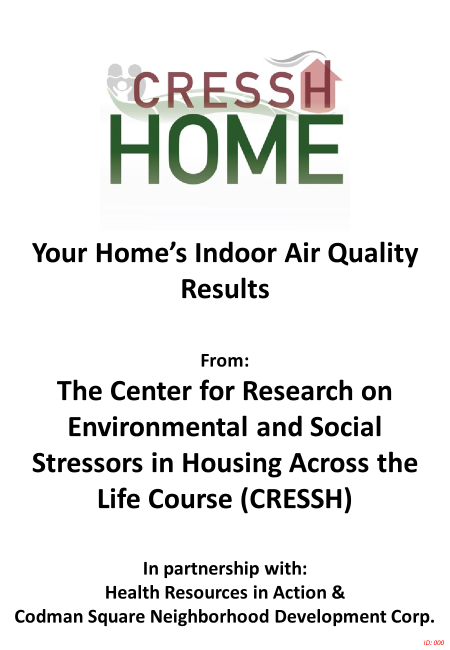


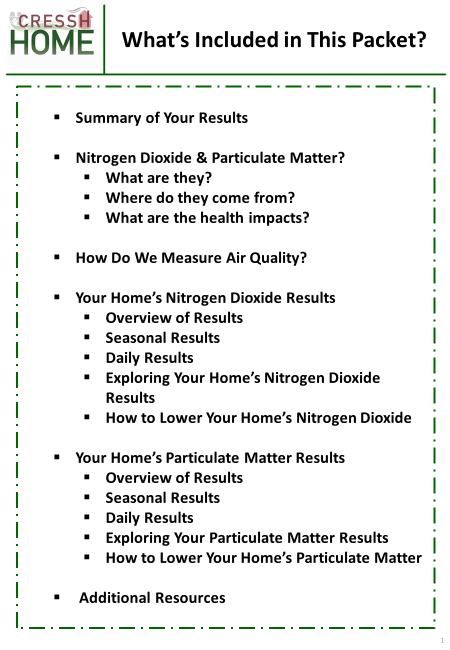


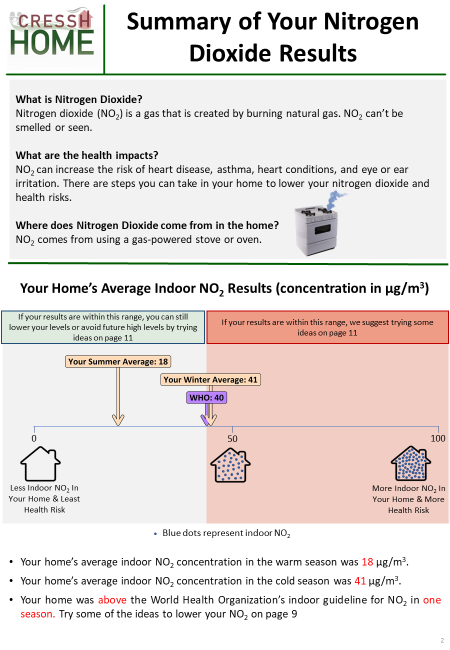


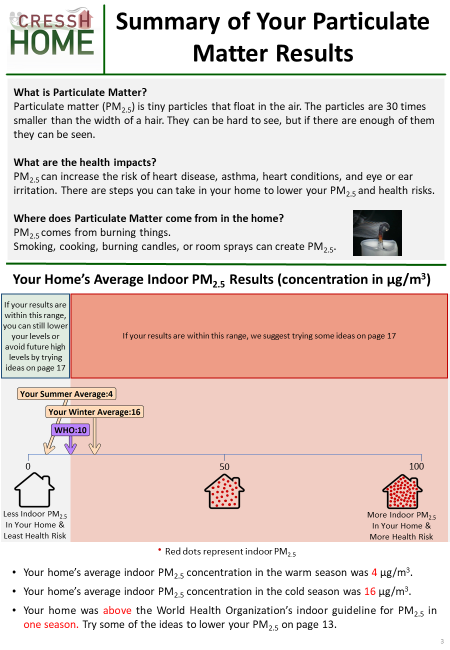


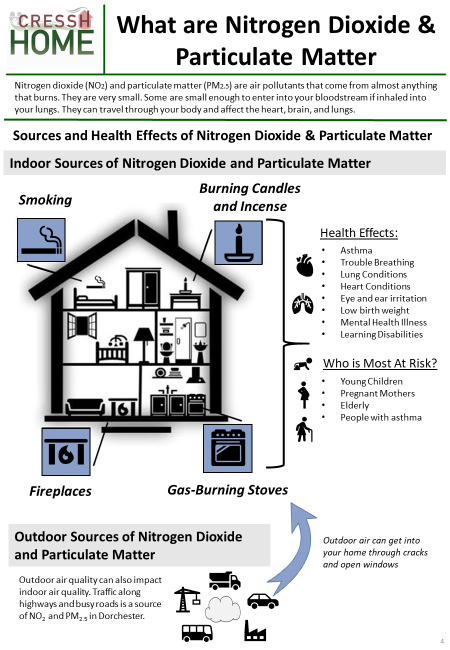


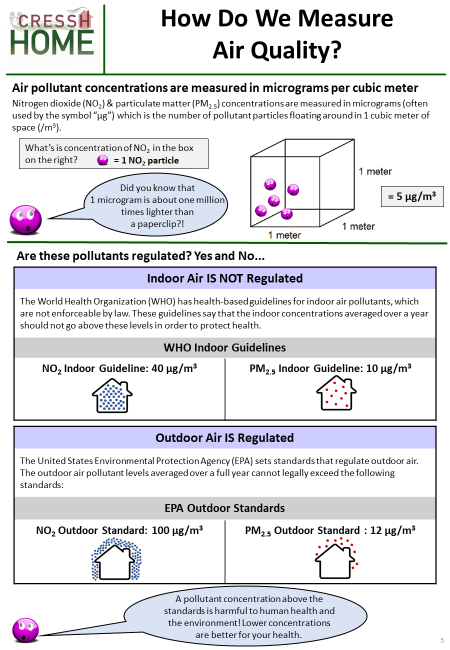


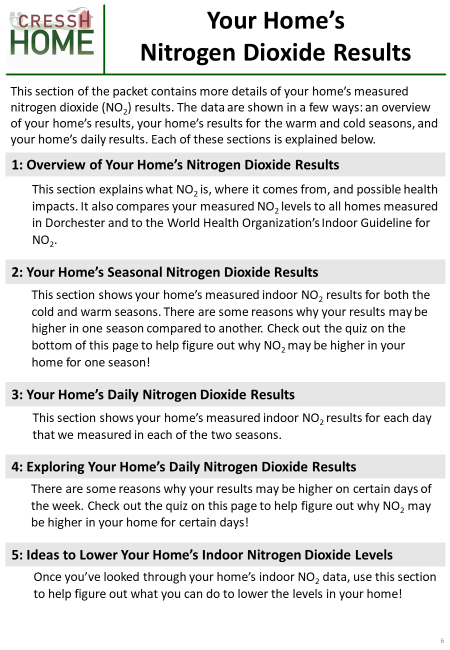


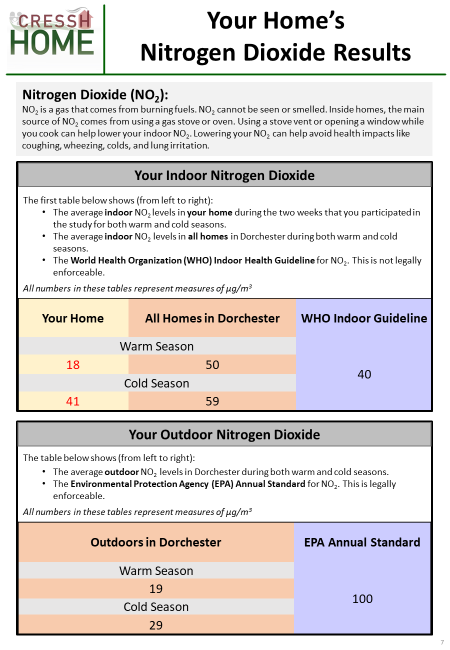


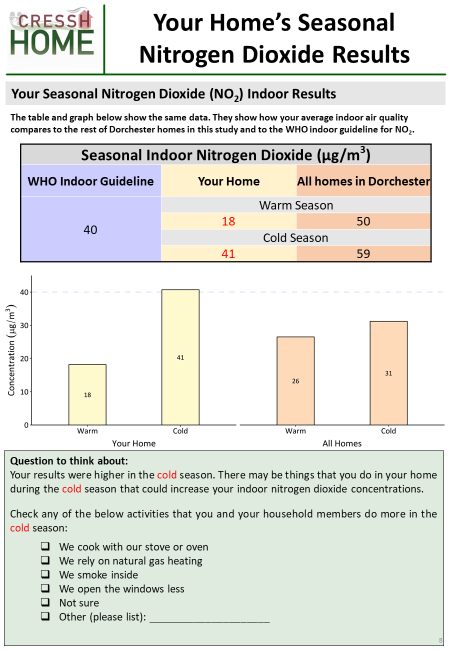


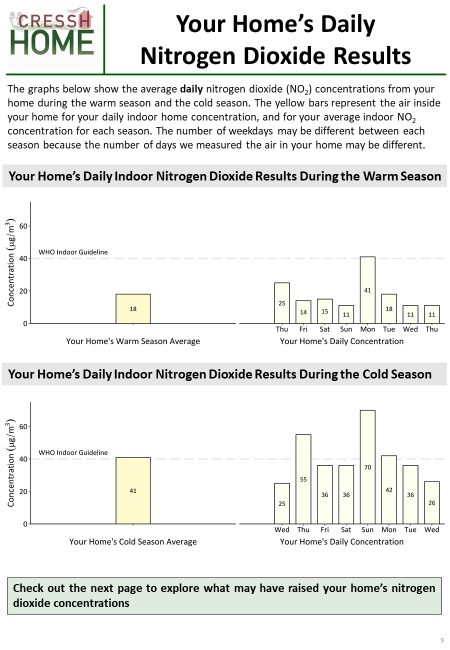


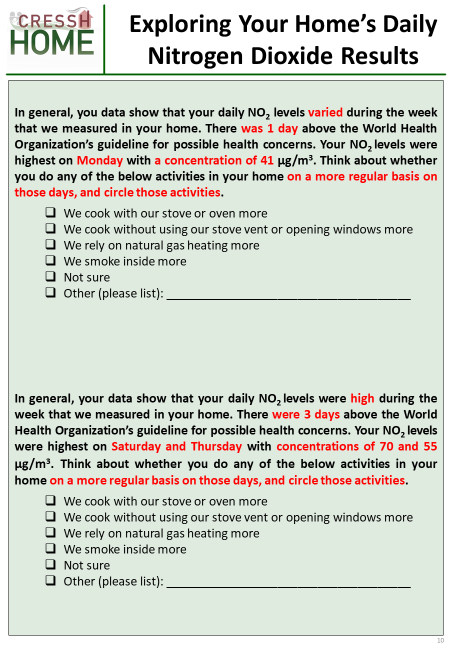


#
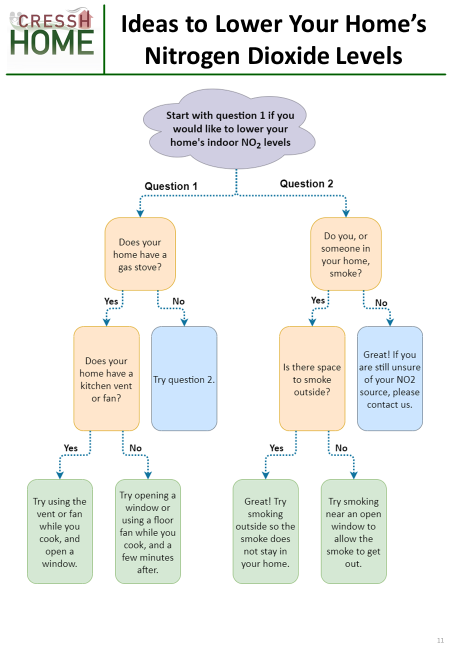


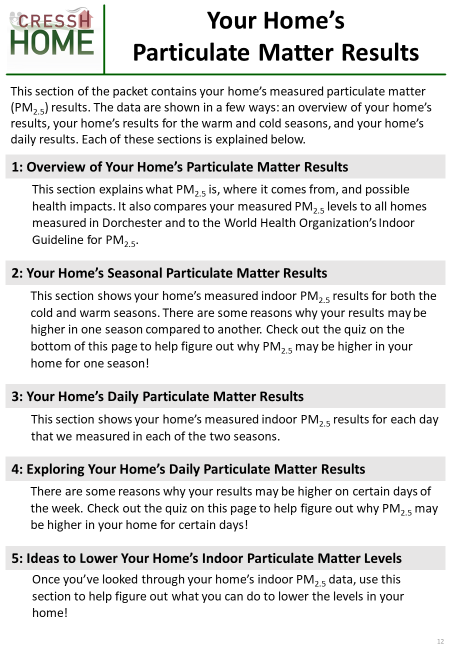


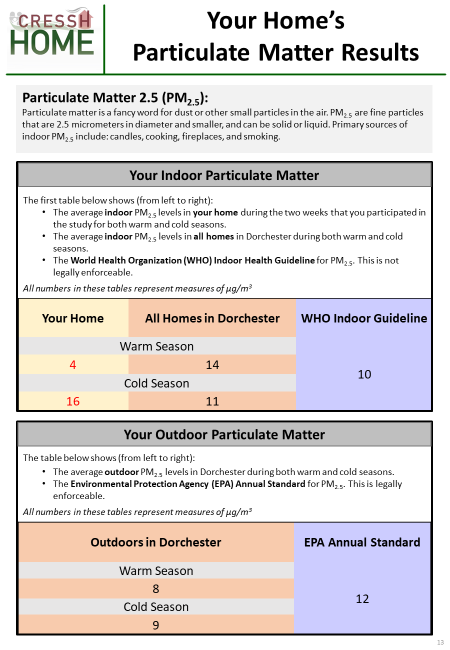


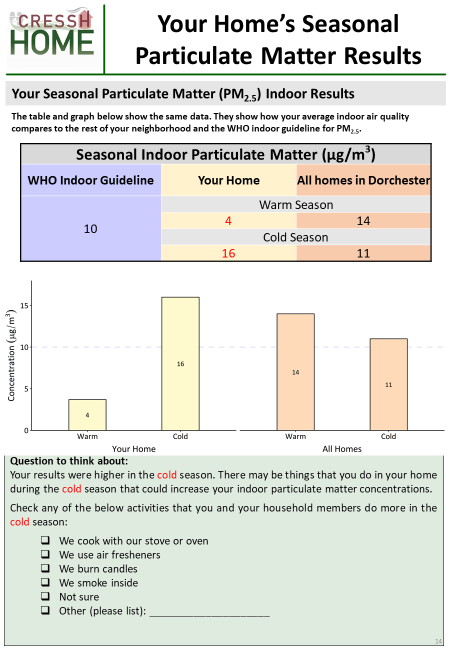


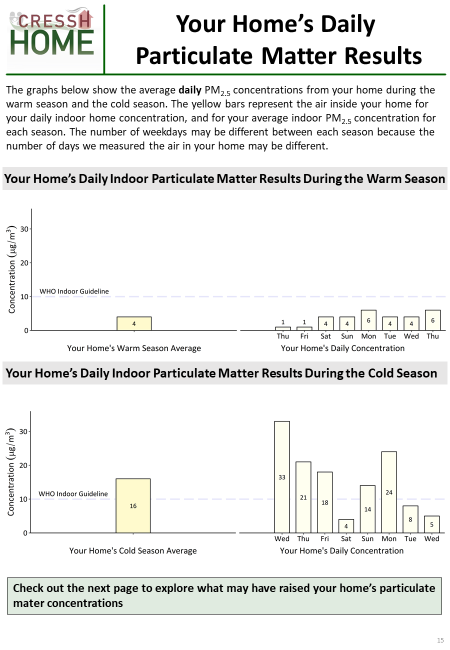


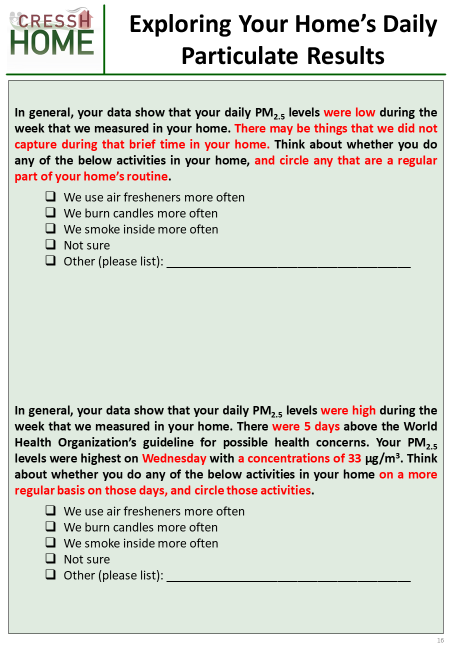


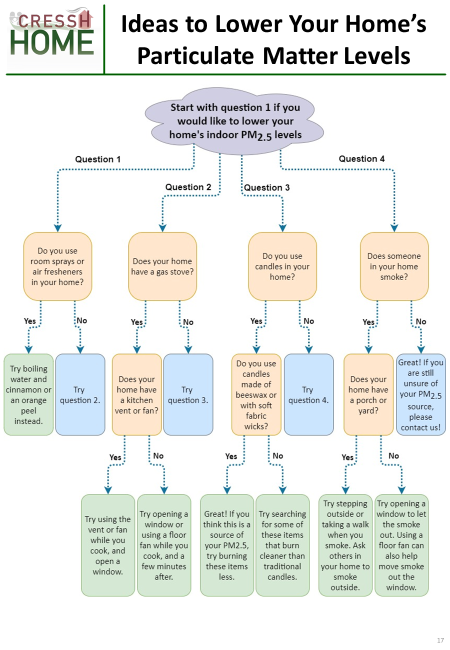


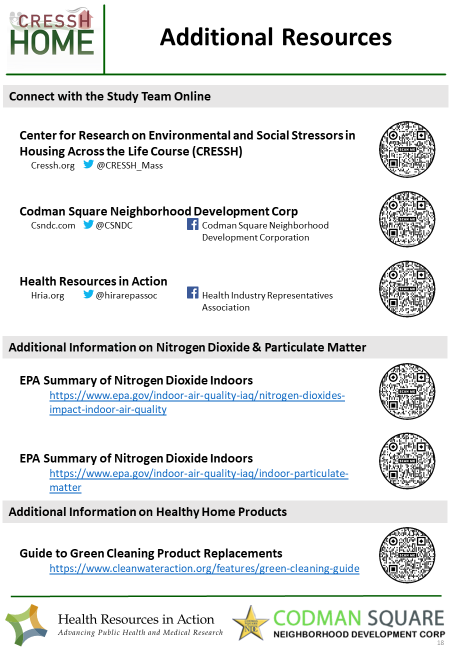


1. E. Polka *et al.*, “MCR : Open-Source Software to Automate Compilation of Health Study Report-Back,” vol. 18, no. 6104, pp. 1–12, 2021. [↑](#footnote-ref-1)
